# Hierarchical partitions of social networks between rivaling leaders

**DOI:** 10.1371/journal.pone.0193715

**Published:** 2018-03-22

**Authors:** Małgorzata J. Krawczyk, Krzysztof Kułakowski, Janusz A. Hołyst

**Affiliations:** 1 Faculty of Physics and Applied Computer Science, AGH University of Science and Technology, al. Mickiewicza 30, 30-059 Cracow, Poland; 2 Faculty of Physics, Warsaw University of Technology, ul. Koszykowa 75, 00-662 Warsaw, Poland; 3 ITMO University, Kronverksky av. 49, RU197101 Saint Petersburg, Russia; University of Oxford, UNITED KINGDOM

## Abstract

A model algorithm is proposed to imitate a series of of consecutive conflicts between leaders in social groups. The leaders are represented by local hubs, i.e., nodes with highest node degrees. We simulate subsequent hierarchical partitions of a complex connected network which represents a social structure. The partitions are supposed to appear as actions of members of two conflicted groups surrounding two strongest leaders. According to the model, links at the shortest path between the rival leaders are successively removed. When the group is split into two disjoint parts then each part is further divided as the initial network. The algorithm is stopped, if in all parts a distance from a local leader to any node in his group is shorter than three links. The numerically calculated size distribution of resulting fragments of scale-free Barabási-Albert networks reveals one largest fragment which contains the original leader (hub of the network) and a number of small fragments with opponents that are described by two Weibull distributions. A mean field calculation of the size of the largest fragment is in a good agreement with numerical results. The model assumptions are validated by an application of the algorithm to the data on political blogs in U.S. (L. Adamic and N. Glance, Proc. WWW-2005). The obtained fragments are clearly polarized; either they belong to Democrats, or to Republicans. This result confirms that during conflicts, hubs are centers of polarization.

## Introduction

The phenomenon of conflict-induced group fission is common in sociology and in life [1–4]. On the other hand, the problem of partition of a network into communities is well-established in the socio-physical literature [5, 6]. As a rule, communities are understood there as subsets of the network more densely connected than the network as a whole. Here we explore another criterion of partition, motivated by selection of leaders [7–9]. The function of leaders is assigned to the nodes with high degrees; this assumption is consistent with the literature [10, 11]. An additional condition is that nearest neighbours of each leader remain attached to him during all stages of the partition. Setting this condition, we are led by ‘the 11-th Law of the Inner Cycle’ by John C. Maxwell: ‘a leader’s potential is determined by those closest to him’ [12], which highlights the validity of ties between a leader and his closest circle. As an additional argument, we can indicate historical examples, when a leader finds a group of supporters who remain faithful to him even after he is defeated [13, 14]. We stress that in the effect considered here the initial distribution of the density of links plays a minor role, and therefore the criteria on the quality of partition [5] do not apply.

The problem we intend to solve is as follows. For a given procedure of separating rival leaders as far as they can appear, we intend to find the size distribution of obtained fragments of the network. The procedure is to divide the network into fragments, centered around local leaders i.e. nodes with highest connections degrees. In each fragment, two main leaders are identified, and the split is simulated again. The algorithm terminates, when each obtained fragment cannot be divided according to the above conditions.

The initial network structure used here is the Barabási-Albert (BA) network resulting from the preferential attachment during the network growth [15, 16]. The distribution of node degrees of BA networks is a power law and it resembles degree distributions observed in many social structures [17–21] (with family trees as one of obvious counterexamples). One has to stress that BA networks can serve only as approximated synthetic models since they do not display several features of real social systems, e.g. values of clustering coefficients for large BA networks are much lower than such values for real social networks of comparable sites [22]. Here we consider inclusion hierarchy of network fragments [23], which appear as a result of subsequent partitions. To simulate the partitions, we need information on degrees and mutual distances of local hubs. On the contrary, as it is noted in the next section, the clustering coefficient of hubs plays no role in the algorithm; therefore the Barabási-Albert structure seems acceptable.

In the next three sections we report our algorithm, numerical results, and analytical evaluations, respectively. The section afterwards is devoted to an application of our algorithm to the data on American political blogs [24]. Discussion and summary are given in the last section.

## The algorithm

A scale-free Barabási-Albert network of *N* nodes is constructed in the standard way with the application of preferential attachment [25]. This means that each new node is attached to *M* previously existing nodes. This defines the attachment parameter *M*, i.e. the number of links from a newly attached node to old nodes. Our calculations are performed for *M* = 1, 2 and 3. Links of the network are numbered with an index *T* according to the order in time; those added later have larger numbers *T*. For links added simultaneously, i.e. with the same node, their mutual order is not relevant. A node is found with the largest degree *k*_*m*_, and it is marked as the first leader. The rival leader is found as the node with maximal degree, less or equal to *k*_*m*_, such that the distance to the first leader is not smaller than three links.

The process of cutting links starts from a selection of the shortest path between the leaders. If there is more than one path, we concentrate on one of them. If the length of the path is exactly three, there is only one link in the middle to be cut. If the shortest path consists of more than three links, the cutting can be performed in two ways; either we select the link with the lowest number *T* (variant A) or the link with the highest number *T* (variant B). Comparing the results of both, we will be able to state, how the selection of the link age is relevant. The process is repeated: again and again the shortest path between the leaders is found and one of the links is cut. We add that the clustering coefficient of a hub does alter neither its distances to other nodes, nor its degree. Also, bonds between neighbors of a hub are never cut in the splitting procedure. Therefore, for our purposes the low clustering of BA structures should not be an obstacle.

When the network is split into two, there is one leader in each fragment. For each of them we appoint a new rival leader with the same method as above. Then, the procedure of splitting is repeated. If for all nodes, the shortest path from a node to the leader is less than three links, the algorithm is stopped. Computationally, the procedure is comparable to the min *s* − *t* cut problem: how to cut a minimal number of edges as to separate node *s* from node *t* [26]. The method is to remove edges along the shortest path between *s* and *t* nodes. This can be seen as a variation of the divisive algorithm of Girvan and Newman [27], who generalized the definition of betweenness centrality of nodes to edges. We note that the *s* − *t* centrality of nodes has also been used in literature [28]. It is known that the min *s* − *t* cut problem is solvable in polynomial time [29, 30].

**Fig 1:**
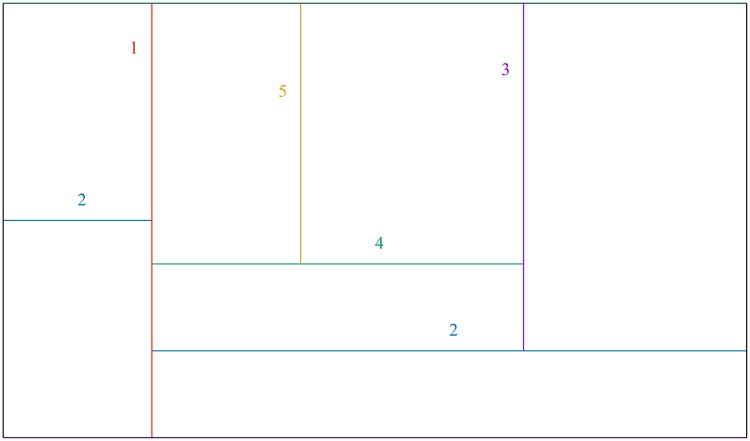
Subsequent divisions of the network lead to the levels of a hierarchy. For the sake of clarity, here the same idea is shown for a rectangle. Boundaries of areas resulted from subsequent partitions are marked with numbers from 1 to 5, and by color (online): red, blue, black, dotted green, dotted yellow. Boundaries made earlier are supposed to be thicker. In this example, the system is divided into two, next into four. In further steps, only one fragment is being divided.

During one time step, each fragment of the network which can be divided is divided. In other words, subsequent partitions are performed on each fragment of the network simultaneously. In Fig 1, we show the same idea, pictured for clarity on a rectangle. There, the first partition is marked by a vertical line 1. Each of two parts of the rectangle are divided at step 2—these are two horizontal blue lines marked by 2. One part of the rectangle (upper right) is divided into two by the black vertical line, etc. The final partition shown in the picture is obtained in five time steps. At each time step, the resulting parts are nested in the parent rectangle; that is why we can speak about *a hierarchy of partitions*.

## Numerical results

**Fig 2:**
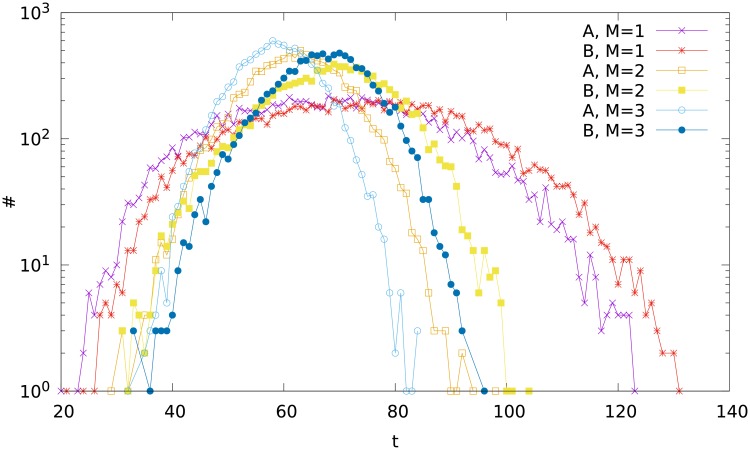
The frequency distribution ♯(*t*) of the time length *t* of the division process for the variants A and B, and for different values of the parameter M.

In Fig 2, we show the frequency distribution ♯(*t*) of the duration time of the separation, measured as the number of the partitions. There is no marked difference between particular cases (different variants A and B, different values of *M*), except the case of large *s* (on the right side of the plot). There we see that basically, the larger value of *M*, the shorter time (i.e. smaller number of divisions). This result is a consequence of the fact, that for a more dense network (larger *M*), the fragment of a diameter three—which cannot be divided anymore—has more nodes, and therefore the time of division to reach such a fragment is shorter. In other words, the division of a denser network stops earlier.

On the other hand, the times obtained in the variant A are usually shorter than those in the variant B. Recall that, as explained in the section *The algorithm*, in the variant A we cut at first the links which are formed earlier. Then, our result means that cutting the links which are formed earlier is more efficient. This result is closely akin to the fact, that the scale-free networks are more sensitive to the removal of nodes with larger degree [31]. By means of the standard algorithm of growth of scale-free networks, these nodes are older, i.e., they have been added earlier.

**Fig 3:**
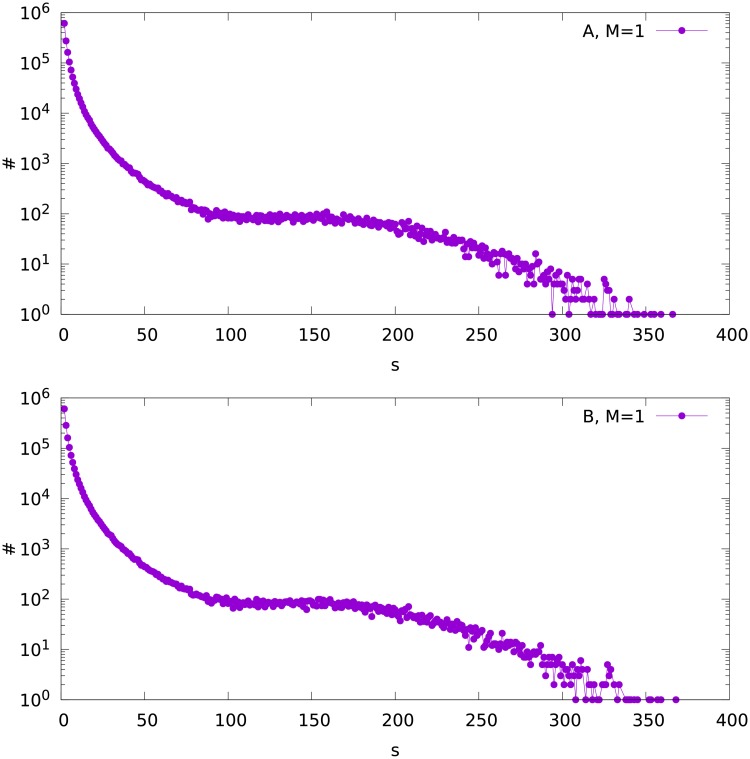
The frequency distribution ♯(*s*) of the fragment size *s* for variant A (upper plot) and B (bottom), for the attachment parameter *M* = 1.

**Fig 4:**
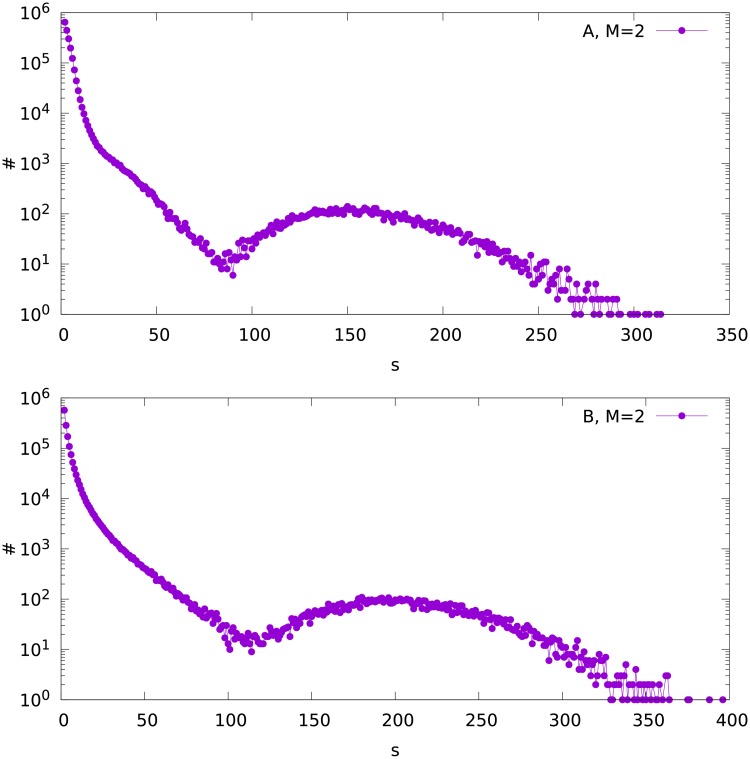
The frequency distribution ♯(*s*) of the fragment size *s* for variant A (upper plot) and B (bottom), for the attachment parameter *M* = 2.

**Fig 5:**
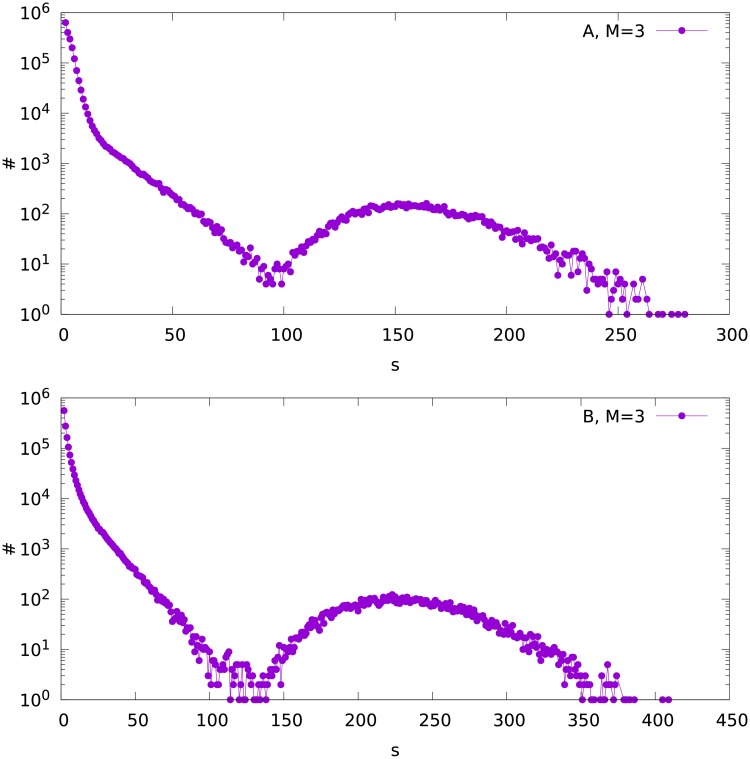
The frequency distribution ♯(*s*) of the fragment size *s* for variant A (upper plot) and B (bottom), for the attachment parameter *M* = 3.

The calculations have been performed for *N* = 1000. For both variants A and B and *M* = 1, 2 and 3, the size distributions of the fragments of the network for the obtained final partition are shown in Figs 3, 4 and 5. It can be seen that in all cases, the distribution consists of two parts. The maximum on the right side of the plot (for large *s*), visible in the semilogarithmic scale, shows the largest component which contains the node with the largest degree in the whole network. The volume of this peak is therefore equal to the number of simulated networks, which is *K* = 10^4^. We expect that the largest fragment contains at least the hub plus its nearest neighbours. Accordingly, *K* largest sizes of the network fragments can be used to evaluate numerically the size of the largest component. We shall also estimate analytically the size of this component in the next section.

**Fig 6:**
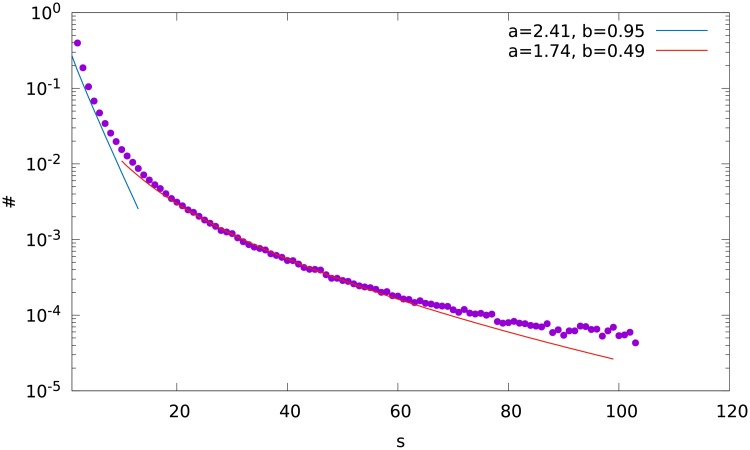
An exemplary fit of the numerical results by the Weibull distribution for M = 1, variant B. The labels *a*, *b* are the parameters of the Weibull distribution.

On the left side of the Figs 3, 4 and 5 (small *s*) we see the size distribution of the remaining fragments of the network. We can analyze this part independently on the maximum on the right side of the plots. Trying to fit these left parts of the plots with the two-parameter Weibull distribution [32]

$$\begin{array}{c} \rho \left(s\right)=ab{\left(as\right)}^{b-1}e^{-{\left(as\right)}^{b}} \end{array}$$ (1)

we observe two ranges of *s* where different values of the parameter *b* are obtained. For each out of the six (*M* = 1, 2 and 3, variants A and B) distributions, *b* is about 0.95 for low *s*, and about 0.4-0.5 for higher *s*. The ranges of size *s* where the different values of *b* fit can be seen in Figs 3, 4 and 5. The parameter *a* varies between 0.4 and 2.8. An exemplary fit is shown in Fig 6.

## Analytical estimation of mean size of giant subtree

We observe that right parts of the distributions in Figs 3, 4 and 5 correspond to the network fragments that include the main hub of the system. Let us estimate the *mean size* of this component (we call it ‘a giant subtree’ here) using a simple mean-field approach. Following [15, 33–36] the degree of a node *a* in Barabási-Albert network in mean-field approximation increases with time *t* as

$$\begin{array}{c} k_{a}\left(t\right)=M\sqrt{t/t_{a}} \end{array}$$ (2)

where *t*_*a*_ is the time when the node *a* was attached to the network.

Let assume for the simplicity that *M* = 1, thus the network is a loopless tree. The idea is to count the seed cluster, its nearest neighbours and their neighbours with the condition that the latter have no other neighbours; they remain as leaves. In other words, the giant subtree contains the seed, all its children, and those grandchildren that have not got their own offspring. This means that the giant subtree includes only the grandchildren that are not too mature. Let *Ω*_*h*_ stands for a *h*− sphere of the seed, i.e. it is a set of all nodes that are at a distance *h* = 0, 1, 2… from the seed nodes. Following the assumed splitting algorithm the giant subtree consists of spheres *Ω*_0_ (the seed), *Ω*_1_ (its children), and a part of nodes from *Ω*_2_ (grandchildren) that are not connected to any node in the sphere *Ω*_3_. Let at time moment *t* = 1 a pair of connected nodes—the seed—started the network evolution as in Barabási-Albert model [33]. Taking a pair as the seed, we get that degree of each those nodes is one at time *t* = 1, in accordance with Eq 2. Each of these two initial nodes will become a network hub, i.e. a node with the highest degree. Since the hub’s degree is $h_{hub}\left(t\right)=\sqrt{t}$ thus at moments *t*_*i*_ = *i*^2^ where $i=1,2,3,\ldots ,\leq \sqrt{t}$ nodes *i* in *Ω*_1_ emerge. Note that this reasoning *underestimates* the size of the giant subtree. In fact, it assumes that the hub is not increasing at all at time moments *t* = 2, 3, although the evolutionary algorithm of the Barabási-Albert network works at these times as well. Following (2) degrees of nodes from the sphere *Ω*_1_ increase as

$$\begin{array}{c} k_{i}\left(t\right)=\sqrt{t}/i \end{array}$$ (3)

Let the pair of indices (*i*, *j*) label nodes in sphere *Ω*_2_ directly connected to a node *i* in the sphere *Ω*_1_. Nodes (*i*, *j*) emerge at moments *t* = *t*_*i*,*j*_ = *i*^2^*j*^2^, $j=2,3,4,\ldots ,\leq \sqrt{t}/i$ (let us note that *t*_*i*,1_ corresponds to the emergence of the node *i*). Following (2) degrees of these nodes increase as

$$\begin{array}{c} k_{i,j}\left(t\right)=\sqrt{t}/\left(ij\right) \end{array}$$ (4)

Let the triple (*i*, *j*, *l*) label a node in the sphere *Ω*_3_ that is directly connected to the node (*i*, *j*) from *Ω*_2_. It follows from Eq (4) that the degree of the node (*i*, *j*) equals to *k*_*i*,*j*_(*t*) = 2 at *t* = 4*i*^2^*j*^2^. It means that at the moment *t*_*i*,*j*,2_ = 4*i*^2^*j*^2^ there is already one node (*i*, *j*, 2) in *Ω*_3_ that is directly connected to the node (*i*, *j*). Thus if *t* ≥ 4*i*^2^*j*^2^ then the node (*i*, *j*) is not in the same subtree as the hub when our splitting algorithm is completed. It means that among all nodes (*i*, *j*) only nodes described by pairs of labels such that $i=1,2,3,\ldots \leq \sqrt{t}$, $\sqrt{t}/\left(2i\right)\leq j\leq \sqrt{t}/i$ and *j* ≥ 2 are in the same subtree as the hub. When *t* ≫ 1 the number of such nodes from the sphere *Ω*_2_ can be approximately expressed by the integral

$$\begin{array}{c} S_{2}^{hub}\approx \int_{1}^{\sqrt{t}}di\int_{\sqrt{t}/\left(2i\right)}^{\sqrt{t}/i}dj=\sqrt{t}\ln\left(t\right)/4 \end{array}$$ (5)

The above formula does not take into account the condition *j* ≥ 2 thus the number of nodes is overestimated. For large networks when *t* ≫ 1 we can estimate the size of the subnetwork with the hubs as

$$\begin{array}{c} S_{total}^{hubs}\left(t\right)\approx 2\left(1+\sqrt{t}+\sqrt{t}\ln\left(t\right)/4\right) \end{array}$$ (6)

**Fig 7:**
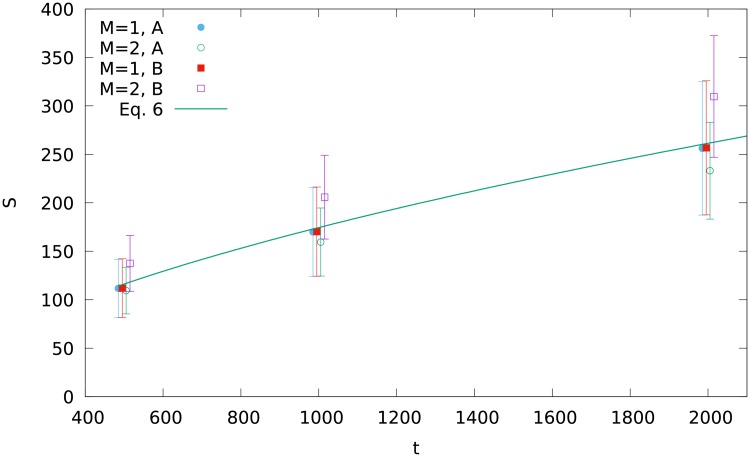
Numerical results on the size of the network fragment with the largest hub, for *t* = 500, 1000 and 2000, *M* = 1 and *M* = 2, for both variants A and B. The continuous line is the prediction of Eq 6.

For *t* = 1000 we get $S_{total}^{hubs}\approx 2\left(1+32+56\right)=178$. This value is not far from the maximum observed in Fig 3 for $S_{numer}^{hub}\approx 170$. The numerical values of $S_{total}^{hub}\left(t\right)$ for *t* = 500 and 2000 are found to be about 112 and 256, respectively. In this sense numerical values of $S_{total}^{hub}\left(t\right)$ scale with the network size *t* similarly as the formula (6), which gives the results 115 and 257, respectively. Similar proportionality is obtained for *M* = 2, as shown in Fig 7 and in *Supporting Information*, although the differences between the results for the variants A and B are much larger there.

## Application to the network of blogs

In model social networks, leadership is often represented by the high degree of a node; this representation finds its support in real data [10, 11]. Here we use this analogy to apply our algorithm to the network of political weblogs in U.S., carried on within two months before the presidential election in 2004 [24]. There, nodes are blogs and a link means that one blog refers to another. The blogs are tagged as democratic or republican. Our intention is not as to check if they are politically oriented, because clearly they are, but rather to verify if our algorithm based solely on the network topology can reproduce this polarization.

**Fig 8:**
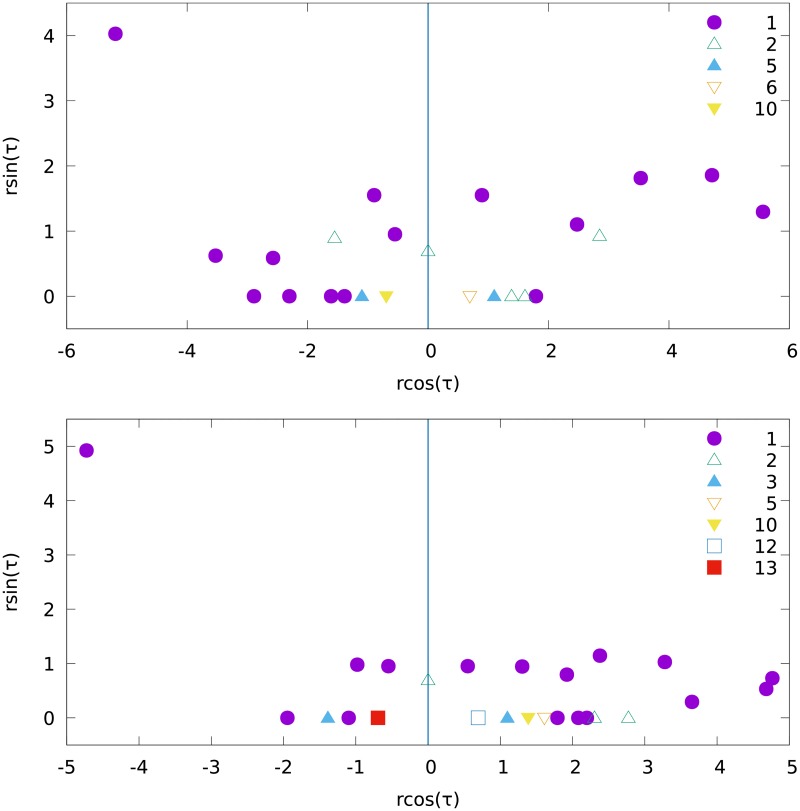
The fragments obtained by the partition of the network of political blogs [24]. The position of a fragment is related to its size and its content: *r* = log(*N*_*R*_ + *N*_*D*_), and Θ = *πN*_*D*_/(*N*_*R*_ + *N*_*D*_), where *N*_*D*_ (*N*_*R*_) is the number of blogs tagged as democratic (republican). The same data on the blogs [24] are used twice, according to the two versions of the algorithm: stronger (weaker) links are cut at first for the upper (the lower) picture. For some positions in the plane, we find more than one fragment, hence different symbols are used for the number of fragments at a given position (see legends on both plots). In both figures, the blue dot on the upper left corner contains the largest hub.

For our purposes, links are symmetrized. Yet, if two blogs refer to each other, the weight of their mutual link is 2 (a bidirectional reference); otherwise it is either 1 (an unidirectional reference) or zero (no reference). These weights allow to perform the simulation in two ways, cutting stronger or weaker links at first, as we previously did with younger or older links. In Fig 8 the distribution of sizes and political orientations of the obtained fragments are depicted for both versions of the algorithm.

As we see in Fig 8, the majority of fragments is fully polarized, i.e. either the number *N*_*D*_ of blogs tagged as democratic, or the number of blogs *N*_*R*_ tagged as republican in a given fragment is equal to zero. This corresponds to the value of the parameter Θ = *πN*_*D*_/(*N*_*R*_ + *N*_*D*_) either zero or *π*. Even the largest fragments are clearly polarized; their partition shows a clear majority of democratic blogs. Concluding, in all but some fragments, this or that political orientation prevails. It is also worthwhile to note that the nodes which play the role of leaders (hubs) at first step of separation belong to different parties in both variants of the algorithm. Also, the fragments which contain these hubs are the largest ones. In the largest fragment which contains the largest hub, on the upper picture: *N*_*D*_ = 684, *N*_*R*_ = 236, and on the lower picture: *N*_*D*_ = 564, *N*_*R*_ = 150. In the second largest fragment, which contains the second largest hub, in the upper picture: *N*_*D*_ = 6, *N*_*R*_ = 118, and in the lower picture: *N*_*D*_ = 22, *N*_*R*_ = 280. We note that in the absence of polarization, the most probable partition would be *N*_*D*_ ≈ *N*_*R*_.

## Discussion

Our simulations are performed for two versions of the algorithm, where old or new links are cut. Yet, there are no qualitative differences between the results. We deduce that any intermediate version of the algorithm should produce similar results; in other words, the order of cutting links is not that crucial. The same conclusion applies also to the order of cutting, uni- or bidirectional, in the network of blogs.

The observed distribution of sizes of split components consists of two parts. Smaller components follow the Weibull distribution, known also as the generalized Mott distribution, that has been applied to describe the size distribution of the fragments of explosive warheads [37]. The difference of the parameters *b* of the Weibull distributions we have found in different ranges of the fragment size *s* indicates that small fragments are produced according to a different rule than larger ones. We deduce that these small fragments come from the surface of the largest fragment containing the first leader. Their particular distribution is due to the condition that the nearest neighbours of the leader remain attached to him. Largest fragments follow from the structure of the closest neighbourhood of the main network hub or the group primary group leader. Our numerical simulations as well as mean-field theory show that size of the largest fragment scales approximately as the square root of the initial network size.

The results of the application of the algorithm to the network of politically polarized blogs confirm that the obtained network partition properly reflects the conflict, encoded in the network structure. The hubs and their direct neighbourhoods belong to the same political orientation in most cases. We treat this result as the validation of the algorithm of the network partition.

As the phenomenon of conflict-induced group fission is common in social life, it is natural to analyse possible conflicts and their expected consequences. Hence, a prudent leader tries in advance to set his opposition at variance; two weak rivals are better than a strong one. This strategy is known throughout the whole human history [38–40]. Our considerations can be seen as a mathematical illustration of importance of this scenario.

## Supporting information

S1 Table The mean value of the size of the largest component is given, as dependent on the network initial size *N* and on the growing parameter *M*. (EPS)

S1 Fig The frequency distribution ♯(*s*) of the fragment size *s* for variant A (upper plot) and B (bottom), for *N* = 500 and *M* = 1. (EPS)

S2 Fig The frequency distribution ♯(*s*) of the fragment size *s* for variant A (upper plot) and B (bottom), for *N* = 500 and *M* = 2. (EPS)

S3 Fig The frequency distribution ♯(*s*) of the fragment size *s* for variant A (upper plot) and B (bottom), for *N* = 2000 and *M* = 1. (EPS)

S4 Fig The frequency distribution ♯(*s*) of the fragment size *s* for variant A (upper plot) and B (bottom), for *N* = 2000 and *M* = 2. (EPS)
